# Enhancing Neuroplasticity Post Stroke: The Role of Cognitive–Behavioral Training

**DOI:** 10.3390/brainsci15040330

**Published:** 2025-03-22

**Authors:** Mohamed Rasmy Moursy, Abdulalim A. Atteya, Hoda M. Zakaria, Zizi M. Ibrahim, Olfat Ibrahim Ali, Nouf H. Alkhamees, Mye A. Basheer, Noura A. Elkafrawy

**Affiliations:** 1 Department of Physical Therapy, Akhmim Hospital, Sohag 82749, Egypt; termazy2040@gmail.com; 2 Department of Physical Therapy for Neurology, Faculty of Physical Therapy, Cairo University, Giza 12613, Egypt; abdulalima.atteya@gmail.com (A.A.A.); dr.hodazakaria@cu.edu.eg (H.M.Z.); noura.elkafrawy@cu.edu.eg (N.A.E.); 3 Department of Rehabilitation Sciences, College of Health and Rehabilitation Sciences, Princess Nourah bint Abdulrahman University, P.O. Box 84428, Riyadh 11671, Saudi Arabia; zmibrahim@pnu.edu.sa (Z.M.I.); nhalkhamees@pnu.edu.sa (N.H.A.); 4 Physical Therapy Program, Batterjee Medical College, Jeddah 21442, Saudi Arabia; 5 Basic Science for Physical Therapy, Faculty of Physical Therapy, Cairo University, Giza 12613, Egypt; 6 Department of Neurology and Clinical Neurophysiology, Faculty of Medicine, Cairo University, Giza 12613, Egypt; myebasheer@hotmail.com

**Keywords:** stroke, cognitive–behavioral training, cognitive impairment, cortical reorganization

## Abstract

**Background**: Stroke is a primary cause of adult disability and often causes cognitive impairment. Rehabilitation interventions aim to enhance patients’ cognitive abilities, thereby addressing care needs, improving quality of life, and optimizing performance in compromised functions. **Objective**: To evaluate the impact of incorporating cognitive–behavioral training (CBT) into a selected exercise program on cortical reorganization and cognitive recovery in post-stroke patients. **Methods**: Thirty post-stroke patients of both sexes (27 male and 3 female) aged from 40 to 65 years were randomly divided into two groups: the study group (n = 15) received CBT combined with a selected exercise program including weight-bearing, balance, and aerobic exercises, while the control group (n = 15) underwent the selected exercise program only. All participants engaged in an 8-week intervention with three sessions per week. Cortical reorganization was measured using quantitative electroencephalography (QEEG) at electrode sites F3, F4, T5, and T6, and cognitive function was assessed using the Montreal Cognitive Assessment (MoCA) and RehaCom, focusing on memory, attention, concentration, logical reasoning, and reaction behavior. Assessments were carried out for all patients before and after the 8-week treatment program. **Results**: Improvements were assessed through three key measures: QEEG, the MoCA, and RehaCom. Post-intervention, the study group demonstrated a significantly higher (alpha + beta)/(delta + theta) ratio at F3, F4, T5, and T6 (*p* < 0.01), indicative of enhanced cortical reorganization. MoCA scores increased by 16.98% in the study group compared to 7.40% in the control group (*p* < 0.01). Additionally, RehaCom assessments revealed marked improvements in memory, attention, logical reasoning, and reaction behavior in the study group (*p* < 0.01). **Conclusions**: Integrating cognitive–behavioral training with a selected exercise program significantly enhances cortical reorganization and cognitive recovery in post-stroke patients. These findings suggest that adding CBT to rehabilitation protocols can effectively address deficits in memory and attention, ultimately improving functional outcomes.

## 1. Introduction

Because of the rising incidence and decreased mortality associated with stroke, post-stroke cognitive impairment (PSCI) is becoming more common in people after stroke [1]. Cognition encompasses the brain’s core functions for processing, storing, retrieving, and manipulating the information necessary for problem-solving. After a stroke, up to 55% of patients experience deficits in episodic memory, 40% experience executive function impairment, 23% show deficits in language, and 70% suffer some cognitive decline, all of which affect their functional abilities, work performance, and capacity for independent living [2]. PSCI significantly affects independence and the ability to return to work [3].

Memory, learning, and attention problems can have a substantial impact on a stroke survivor’s functional independence, and multiple studies have found that higher levels of cognitive impairment are linked to lower self-reported quality of life [4]. These associations have prompted significant efforts to identify effective treatments to improve cognitive function following a stroke [5].

Computerized cognitive training consists of organized exercises on standardized, mentally stimulating tasks [6], offering several benefits compared to traditional drill-and-practice approaches. These advantages include engaging visual interfaces, efficient and scalable delivery, and the ability to continuously adjust training content and difficulty based on individual performance [7].

The RehaCom software package offers a comprehensive approach to cognitive assessment and rehabilitation. This evidence-based tool integrates three core therapeutic strategies: enhancing patients’ understanding of cognitive processes, boosting motivational aspects, and developing compensatory techniques and adaptive skills to manage cognitive deficits [8].

Neurorehabilitation aims to directly quantify brain damage healing through the use of trustworthy, objective, and interpretable measurements of neuroplasticity or changes in brain function [9]. Because it measures cortical activity and reflects the brain’s spatiotemporal information, quantitative electroencephalography (QEEG) is a popular tool for developing assistive rehabilitation devices and evaluating neurophysiological responses to rehabilitation interventions. QEEG is also a non-invasive and easy way to record brain activity. QEEG signals are recorded from four standard frequency bands, alpha (8–12 Hz), beta (12–30 Hz), theta (4–8 Hz), and delta (1–4 Hz) waves, providing valuable insights into cortical brain activity [10]. It is cheap, easy, and nearly risk-free when compared to other brain imaging methods. It offers electrophysiological information that is not available from other imaging modalities or clinical evaluations, and it has a high temporal resolution. Additionally, without requiring the patient to cooperate, QEEG allows doctors to objectively measure brain function and conduct real-time brain evaluations [11].

Several studies have demonstrated the efficacy of cognitive–behavioral training (CBT) in improving cognitive functions such as memory, attention, and executive function in stroke patients. CBT facilitates neuroplasticity by modulating neural oscillations and strengthening synaptic connections, thereby enhancing cortical reorganization. Research has also shown that cognitive training interventions improve functional brain connectivity and contribute to cognitive recovery in stroke populations [12,13,14]. Compared to other cognitive rehabilitation approaches, CBT offers a structured and adaptive method that targets specific cognitive domains essential for post-stroke recovery [15,16].

Despite promising outcomes reported in prior studies, the neurophysiological mechanisms by which cognitive–behavioral training (CBT) enhances cognitive recovery in post-stroke patients remain underexplored. In particular, there is limited evidence regarding how CBT influences cortical reorganization using objective neurophysiological measures. This study addresses this gap by combining CBT with a selected exercise to elucidate its impact on neural oscillatory activity and cognitive function in chronic post-stroke patients. Based on the existing literature, the aims of the study are to evaluate the additive effect of CBT when integrated with a selected exercise program in improving post-stroke cognitive impairment (PSCI) and investigate the neuroplastic changes associated with these interventions using electroencephalography (EEG). We hypothesize that adding cognitive–behavioral training using computerized cognitive training to a selected exercise program will lead to greater improvements for post-stroke patients in terms of cortical reorganization, as evidenced by enhanced QEEG indices, and superior cognitive recovery, as reflected in increased MoCA and RehaCom scores, compared to the selected physical therapy program alone.

## 2. Materials and Methods

### 2.1. Study Design

This controlled, randomized study was conducted at two locations affiliated with Cairo University, the outpatient facilities of the Faculty of Physical Therapy and Al Kasr Al Ainy Hospital’s Faculty of Medicine, between November 2023 and April 2024. Before enrollment, each participant was thoroughly briefed on the study’s objectives and methodology and signed an institutionally approved informed consent form to take part. The ethical committee of the Physical Therapy Faculty at Cairo University approved this study (approval number: P.T.REC/012/004915). In addition, the study protocol was registered on the Pan African Clinical Trial Registry with ID: PACTR202401884919252.

### 2.2. Participants

Fifty-two chronic stroke patients underwent eligibility screening for this study. The patients were diagnosed by a neurologist, with CT and/or MRI brain scans confirming the diagnosis.

**Figure 1:**
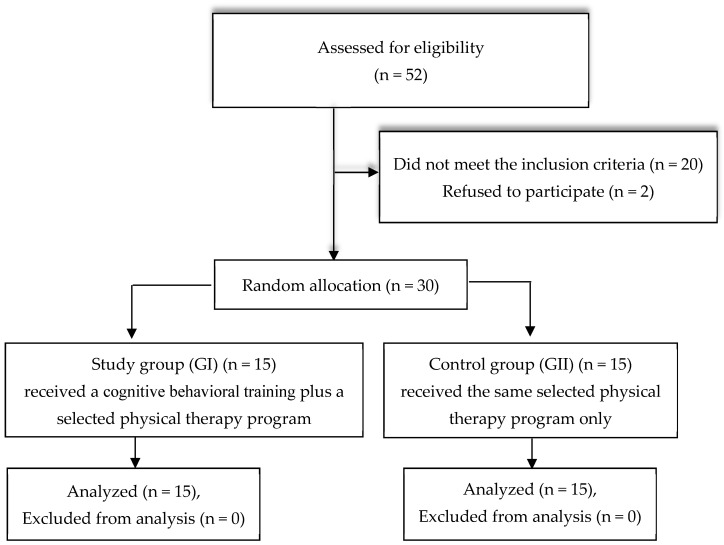
Flow chart showing recruitment of the study participants.

After screening, thirty patients met the inclusion criteria, as shown in Figure 1. The study’s inclusion criteria were as follows: (i) individuals who have suffered an ischemic stroke; (ii) highly educated participants aged between 40 and 65 years, of both sexes; (iii) time since stroke from 6 to 18 months; (iv) individuals exhibiting mild cognitive impairments in areas such as memory, attention, orientation, perception, and executive function, as assessed based on the Montreal Cognitive Assessment (score 18–25); (v) spasticity ranging from (1/1+) according to the Modified Ashworth Scale; and (vi) degree of muscle strength in paretic limb muscles not less than grade 3 according to group muscle test.

Patients were excluded if they (i) had suffered recurring stroke incidents; (ii) presented with hemiparesis resulting from neurological conditions other than stroke; (iii) had a metal implant in their heads; (iv) had a cardiac pacemaker; (v) were medically unstable or uncooperative; or (vi) had aphasia.

The participants were randomly allocated into either the study or the control group. Randomization was carried out by an independent researcher who was not involved in the assessment or in the participants’ treatment. A computer-generated random number table was employed for randomization, and a sealed opaque envelope was utilized for allocation concealments. The study group comprised 15 individuals who received a cognitive–behavioral training (CBT) protocol in addition to a selected physiotherapy program, and the control group consisted of 15 participants who only engaged in the same selected physiotherapy program.

The present sample size was sufficient according to the calculated partial eta squared for the interaction, group, and time effects, which were 0.98, 0.93, and 0.99, indicating a large effect. Based on an effect size of 0.4, alpha level of 0.05, and power of 80%, the estimated sample size was 28 participants in both groups using G*power software 3.1.9.7(University of Düsseldorf in Düsseldorf, Germany).

### 2.3. Outcome Measures

All outcome measures were assessed by a blinded assessor for all patients before and after receiving eight weeks of the assigned treatment program.

#### 2.3.1. Primary Outcome

##### Quantitative Electroencephalography (QEEG)

QEEG is a valid and reliable method to assess brain activity [17]. In this study, we used a QEEG device (EB, Florence, Italy; Mizar—PC Peripheral System CE Version—B9800037800) in brain labs at Kaser Al Aini Teaching Hospital and Cairo University to record the participants’ brain activity. During the recording, the patient wore a comfortable cap while the clinician recorded the QEEG on a computer. To decrease interference from eye movements, blink artifacts, and visual cues, the patients were asked to close their eyes. Data collection followed the International 10–20 system using Ag/AgCl electrodes and a unipolar montage, maintaining impedance below 5 kohm to prevent polarization impact. The brain activity analysis utilized the mean frequency ratio equation of [delta + theta)/(alpha + beta)], measured before and after the program for both groups [18]. Electrode sites F3, F4, T5, and T6 were selected due to their critical roles in post-stroke recovery. F3 and F4 (frontal cortex) are associated with executive function and working memory, while T5 and T6 (temporal cortex) are involved in memory processing and language. Prior research has linked neural oscillatory changes in these regions to cognitive and motor recovery. These sites are commonly used in QEEG studies assessing stroke-related neuroplasticity [19,20].

#### 2.3.2. Secondary Outcome

##### Cognitive Functions

Montreal Cognitive Assessment (MoCA)

The MoCA is a widely adopted cognitive evaluation instrument, particularly effective in detecting cognitive impairments. This 30-point assessment, administrable in approximately 10 min, offers a comprehensive evaluation of diverse cognitive functions, involving short-term memory, visual-spatial abilities, executive functioning, attention, concentration, working memory, linguistic skills, and spatiotemporal orientation. In post-stroke cognitive evaluations, the MoCA has demonstrated superior reliability, sensitivity, and practicality compared to other screening instruments, including the Mini-Mental State Examination [21].

RehaCom

RehaCom, developed by Schubfried (Model NO. 454V, D 14482 Potsdam, Karl-Liebknecht, Austria), offers a sophisticated computer-based cognitive rehabilitation platform. This system functions as an impartial evaluator, providing objective performance feedback and error-specific guidance. Its auto-adaptive algorithms dynamically adjust task complexity to match the patient’s current capabilities, ensuring an optimal challenge level that promotes motivation and engagement. This approach not only enhances therapeutic efficacy but also boosts patients’ self-confidence, potentially reducing common post-brain injury complications such as depression or diminished self-esteem. RehaCom saves all assessment results, allowing for continuous monitoring and prompt adjustment of parameters. The software encompasses thirty-two distinct assessment tasks targeting attention, memory, and executive function, with higher scores indicating superior performance. The computer stores a record of each patient’s performance and progress, enabling easy tracking and monitoring [22]. Implemented via a standard PC with a 19-inch display, a specialized RehaCom panel, and EN/ISO-13485 certified software (1990–1997) [23], the system facilitates the administration of a set of neuropsychological tests, including assessments for attention, concentration, and logical reasoning.

### 2.4. Intervention

The participants in both groups received twenty-four intervention sessions of one hour each, three times weekly over an eight-week period. The control group underwent a selected physiotherapy program, while the study group underwent CBT in addition to the same selected physiotherapy program undertaken by the control group.

#### 2.4.1. Selected Exercise Program

The rehabilitation exercise program consisted of aerobic exercise utilizing a stationary bicycle ergometer (Kettler polo S, Model K07960-700, Germany) for 10 min [24]. Exercise intensity was progressively increased up to 60–70% of maximum heart rate (calculated using the Karvonen formula: Target HR = 60–70% [HRmax − HR rest] + HR rest) [25]. Participants maintained a pedal rate of 50–60 rpm, which has been identified as the most comfortable rate for patients of average fitness [26].

The patients also performed weight-bearing exercises for 10 min, including weight-bearing on an affected upper limb from a sitting position and a modified plantigrade position, weight-bearing in the quadruped position, and a bridging exercise [27]. Weight shifting and strengthening exercises included strengthening exercises for both lower limbs as described by Shao et al. [28], in addition to 10 min of balance training from the sitting and standing positions [29].

#### 2.4.2. Cognitive–Behavioral Training

The RehaCom therapy software (Patient enpult (1990–1997) EN/ISO-13485-certified) is optimized to be operated by the RehaCom Panel. This special panel is large, robust, and easy to handle and understand. So, in this study, the RehaCom Panel was used for the ease and convenience of patients. Training in this study focused on the following areas: attention, concentration, figural memory, reaction behavior, and logical thinking. Attention and concentration training includes of 24 levels of difficulty based on the pattern recognition and comparison. The patient must identify which image in a matrix exactly matches a given sample. The images contain different types of objects, including concrete items (such as fruits, animals, and faces), geometric shapes (such as circles, rectangles, and triangles in varying sizes and arrangements), as well as letters and numbers. Figural memory training consists of 9 levels of difficulty and involves multiple tasks per session, each with an acquisition and recognition phase. In the acquisition phase, the patient is shown pictures or words of concrete objects, with the number of items depending on the difficulty level. Once memorized, the patient proceeds to the recognition phase, where they must identify the correct picture or word from stimuli moving across the screen. The patient selects the correct item when it reaches a designated, red-marked area. Logical reasoning training presents a sequence of pictorial and abstract symbols of different shapes, colors, and sizes arranged in a structured pattern. The patient’s task is to continue the sequence correctly. If an incorrect answer is given, feedback is provided on the specific type of mistake (related to shape, color, and/or size). The difficulty level is adjusted by modifying the length and complexity of the sequence. Reaction behavior training comprises 16 levels of difficulty and focuses on developing responses to signals. During the learning phase, objects and their corresponding reaction buttons are introduced. In the training phase, relevant stimuli appear, and the patient must respond within a specific time frame. Additionally, inhibition control is practiced by incorporating irrelevant stimuli that require no response [30,31]. The computer is a neutral observer providing value-free comments on the patient’s performance and giving error-specific feedback if required. A range of feedback modalities can be set in the parametric menu: (1) acoustic feedback; (2) Visual feedback; and (3) Text/Auto stops [30].

The participants who received cognitive training received three sessions per week, on separate days. They were given a 5 min rest between procedures, with two procedures per session—for example, attention concentration training followed by figural memory and reaction behavior training followed by logical thinking.

One in fourteen people may experience mental fatigue and 6% may experience headaches as a result of using the RehaCom system. As patients continue with therapy and gain familiarity with it, they typically see a resolution of their symptoms. No adverse events were documented throughout the experiment.

### 2.5. Statistical Analysis

Data were obtained from both groups at two time points, prior to and following the treatment program, via QEEG, MoCA, and RehaCom. These data were statistically analyzed and compared using SPSS 26 for Windows (IBM SPSS, Chicago, IL, USA). Between-group comparisons of subject characteristics were conducted using unpaired t-tests, while gender and affected side distribution were analyzed with Chi-squared tests. In addition to the independence of observations, the data were checked by drawing box plots and histograms. The Shapiro–Wilk test was used to determine if the data were normally distributed, and Levene’s test was used to determine if the variances between the groups were similar; in addition, the box plot was utilized to assess the homogeneity of variance–covariance matrices (*p* = 0.089), indicating that the assumption was met and sphericity was assumed. A two-way mixed MANOVA was employed to examine within- and between-group effects on QEEG, MoCA scores, and RehaCom outcomes (including logical reasoning, attention and concentration, memory, and reaction behavior). Post hoc analyses were conducted using Bonferroni-corrected multiple comparisons. Statistical significance was set at *p* < 0.05 for all tests.

## 3. Results

### 3.1. Subject Characteristics

The general demographic information displayed non-significant differences (*p* > 0.05) in terms of the mean values for patient age (*p* = 0.72), frequency and percentage of gender (*p* = 0.54), and affected side distributions (*p* = 0.62) between the study group and the control group, as shown in Table 1.

### 3.2. Effect of Treatment on MoCA, Rehacom Results, and QEEG

A significant interaction between treatment and time was shown by the two-way mixed MANOVA (F = 62.54, *p* = 0.001, η^2^ = 0.98). The main effect of time was statistically significant (F = 232.52, *p* = 0.001, η_p_^2^ = 0.99). Treatment had a significant main impact with a *p*-value of 0.001 and a η_p_^2^ of 0.93.

#### 3.2.1. Within-Group Comparison

In the QEEG data analysis, there was a significant increase (*p* ≤ 0.01) in the (alpha+ beta)/(delta + theta) ratio for F3, F4, T5, and T6 in both the study and control groups post-treatment compared with pre-treatment, as shown in Table 2.

Post-intervention assessment using the MoCA demonstrated significant cognitive gains (*p* =0.001) in both groups compared with pre-treatment, as depicted in Table 3.

The RehaCom data analysis revealed notable improvements (*p* < 0.05) in various cognitive functions for both groups following treatment, encompassing logical reasoning, attention and concentration, memory and reaction behavior levels, and correct reactions. There were significant decreases (*p* ≤ 0.01) in incorrect items, MRT, and missed items in both groups post-treatment compared with pre-treatment, as shown in Table 4 and Table 5.

#### 3.2.2. Between-Group Comparison

There was no significant difference between groups pre-treatment (*p* > 0.05). The study group showed a significant increase in the (alpha+ beta)/(delta+ theta) ratio for F3, F4, T5, and T6 compared with the control group post-treatment (*p* < 0.05); see Table 2.

There was a significant increase in MoCA for the study group compared with the control group post-treatment (*p* < 0.01); see Table 3.

RehaCom data analysis showed a significant increase in logical reasoning, attention and concentration, memory, reaction behavior levels, and correct reactions and a significant decrease in incorrect items, MRT, and missed items post-treatment in the study group compared with the control group (*p* < 0.05); see Table 4 and Table 5.

An ANCOVA was performed to check the effects of the intervention while adjusting for the RehaCom pre-treatment values. The results showed that most of the pre-treatment values did not significantly affect the post-treatment scores, indicating that baseline differences had minimal impact on the observed effects.

Among the RehaCom covariates, the pre-treatment values of the logical reasoning level showed a significant effect on the post-treatment scores for the same covariate (F(1, 20) = 13.597, *p* = 0.001, η^2^ = 0.405). Additionally, the pre-treatment reaction behavior level scores affected the post-treatment logical reasoning level scores, F(1, 20) = 17.287, *p* < 0.001, η^2^ = 0.464. The correct reaction pre-treatment value significantly affected the correct reaction scores (F(1, 20) = 6.893, *p* = 0.016, η^2^ = 0.256). Similarly, the pre-treatment MRI had a significant effect on later MRI scores (F(1, 20) = 12.319, *p* = 0.002, η^2^ = 0.381), and the missed item pre-treatment scores significantly influenced post-treatment missed item scores (F(1, 20) = 10.048, *p* = 0.005, η^2^ = 0.334). Finally, the pre-treatment scores for incorrect items significantly influenced the post-treatment incorrect item scores (F(1, 20) = 8.840, *p* = 0.008, η^2^ = 0.307). However, the other covariates did not show significant effects (*p* > 0.05).

It seems clear that some of the pre-treatment values of the RehaCom parameters affected the outcomes, explaining 19.4% to 46.4% of the variance. However, the group effect size remained high across the RehaCom outcome variables (*p* < 0.001, η^2^ > 0.63 for all outcomes), with effect sizes ranging from 63% to 96%. Moreover, most of the RehaCom covariates were not significant (*p* > 0.05), as shown in Table 6.

## 4. Discussion

The driving force behind this research was examining how cognitive–behavioral training (CBT) might affect brain reorganization in stroke survivors. The participants’ ages varied from 40 to 65; people under the age of 65 account for over 38% of all stroke hospitalizations despite the fact that the risk of stroke increases with age [32]. Age is a significant factor in the likelihood of a poor functional outcome following a stroke. Loss of basic cognitive, sensory, and sensorimotor skills and an increased vulnerability to stress are common structural and physiological changes in the brain that are commonly associated with aging [33]. Changes in neuronal plasticity or cellular alterations that directly impact plasticity mechanisms can also contribute to the deterioration of cognitive function associated with aging [34].

QEEG data from the frontal and temporal areas (F3, F4, T5, and T6) were selected to represent cortical reorganization after CBT because the frontal lobe’s roles in cognitive functions such as working memory (WM) and processing speed (PS) are often severely affected by ischemic stroke. Additionally, the temporal lobe plays a key role in creating long-term memories, processing new information, and forming verbal and visual memories [35].

In the current study, a significant increase in the (alpha+ beta)/(delta+ theta) ratio at F3, F4, T5, and T6 was observed in both the study and control groups post-treatment, with the effect in favor of the study group (*p* < 0.01) suggesting cortical reorganization associated with the intervention. In the chronic stage of stroke, the shift toward increased alpha oscillations is particularly significant, since prior research has demonstrated that dominance of theta and beta coherence negatively correlates with clinical improvement during stroke recovery. Notably, Nicolo et al. (2015) found that a shift back toward alpha oscillations is associated with better motor and language outcomes [19]. These findings align with our QEEG results, reinforcing the hypothesis that CBT facilitates cortical reorganization by promoting neural oscillatory patterns linked to functional recovery. CBT likely enhances neuroplasticity by strengthening neural pathways involved in executive function and attention through repeated cognitive engagement and task-specific training [31]. Cognitive training—encompassing structured activities aimed at improving memory, attention, and problem-solving—has been shown to drive neuroplastic changes, which are believed to underlie the observed improvements in cognitive performance. This improvement may be associated with alterations in regional cerebral blood flow (rCBF) resulting from cognitive training [15]. Additionally, reciprocal exercises activate the frontal areas, which play a key role in information processing, motor planning, and the execution of complex motor tasks [36,37]. Furthermore, aerobic exercise has been found to positively impact cognition by increasing cerebral blood flow and reducing the risk of cardio-cerebrovascular disease [38].

The current results are consistent with those of Shinaver and Entwistle, who proposed that computerized cognitive training leads to improvements in general and social cognition, along with verbal and working memory, attention/vigilance, and processing speed. Working memory training relies on neural plasticity, where the brain is activated and adapts by altering neural pathways and synapses. As a result, computerized cognitive training brings about lasting changes in the brain that persist over time [20].

Cognitive–behavioral training has the potential to stimulate temporal cortical processing, particularly within the paralimbic region which is implicated in emotional processing such as evaluating external stimuli and attributing emotional significance. These areas are part of an alarm system that informs about external danger [39]. This agrees with the results of Farrand et al., who reported that CBT has a significant effect on behavior activation, problem resolution, and the transformation of negative thoughts, cognition, and emotions by promoting engagement in meaningful or enjoyable activities [40].

The current results are also in agreement with Ahrens, who noted that CBT can help to reduce depressive symptoms in some individuals following a stroke and is particularly effective in this population. The expectation is that such patients will adopt better thought processes in order to enhance their recovery [41].

Regarding cognitive functions, the present study revealed a significant improvement in MoCA scores for both groups following the intervention, with the study group again demonstrating superior results (*p* < 0.01). Analysis of the RehaCom data indicated a significant reduction in incorrect items, MRT, and missed items within the experimental group relative to the control group after completing treatment (*p* < 0.001). This may be attributed to CBT’s ability to alter the interconnections between various functional networks, particularly in regions associated with cognitive processing and behavioral regulation [42]. The results agree with Batista et al., who noted that individuals with vascular brain injuries can experience cognitive improvements through various CBT interventions, involving name-face pairing exercises to improve social memory and specific memory training protocols designed to enhance recall rather than recognition based on familiarity [12]. This also agrees with the findings of Jiang et al., who noted that attention and response control are key cognitive functions, whereas activities of daily living (ADL) involve an occupational dimension requiring the coordination of various cognitive, sensory, and motor skills [43]. The enhancement of cognitive abilities, including attention and response control, through CBT and tools like RehaCom can also contribute to better outcomes in activities such as visual learning, visual span, as well as visual and auditory continuous performance. Similarly, Lawson et al. reported recent evidence showing that computerized cognitive training and telehealth approaches for remotely providing compensatory memory skills training can enhance memory and attention post stroke [44].

In a study by De Luca et al., stroke-affected individuals were randomly distributed between CBT using virtual reality (VR) and a group that practiced various cognitive tasks. The CBT using VR involved customized tasks targeting memory, attention, and visuospatial skills and reported significant improvements in overall cognitive function (using MoCA) and visual attention in the CBT group, with some benefits persisting at follow-up [16].

Engaging in regular physical exercise, including both resistance and aerobic exercises, can lead to cognitive improvements in people experiencing mild cognitive impairment (MCI) [45,46]. A meta-analysis carried out in 2021 indicated that physical exercise could enhance cognitive abilities in individuals recovering from stroke. These effects, while mild-to-moderate in magnitude, have been observed even in the chronic stroke phase [47].

The findings of the current study agree with those of Ozen [48], who reported that CBT using computer game systems, which is a modern and attractive approach, has a significant effect on cognitive functions. Although conventional physical therapy also demonstrated some cognitive benefits, the incorporation of multimedia and digital tools in cognitive rehabilitation exercises seemed to yield more significant cognitive gains. Quality of life was improved in both groups.

The current study also agrees with previous studies showing that CBT utilizing VR resulted in significantly reduced post-stroke depression when compared to home-based cognitive tasks. Additionally, improvements in attention, spatial awareness, and overall cognitive function were observed exclusively in the CBT group following the intervention [49]. Faria et al. explored the use of comprehensive VR programs designed to target numerous cognitive domains, involving memory, attention, visuospatial skills, and executive functions, within the context of daily activities. Stroke patients were randomly assigned to either a VR-based CBT group or a conventional cognitive training group (involving puzzles, problem-solving, mathematical skills, or memory exercises). Participants in the VR-CBT group were tasked with completing daily activities at various locations within a virtual city, like a post office or a bank. Following a 12-session training period, the VR-CBT group showed significant enhancements in general cognitive function, attentional processes, and verbal fluency. Interestingly, intra-group analyses revealed improvements in visuospatial and executive functions exclusively within the VR-CBT group [50].

Meanwhile, Yeh et al. illuminated the synergistic benefits of integrating aerobic exercise with computerized CBT for improving cognitive functioning in stroke survivors. Additionally, this combination was found to enhance functional ability, psychological state, and overall cardiovascular condition and potentially accelerate neural plasticity [13].

The current research findings have important clinical implications. Integrating cognitive–behavioral training with a selected physical therapy program appears to enhance both cortical reorganization and cognitive function, as reflected by improved QEEG indices and increased MoCA scores. This combined approach has the potential to mitigate cognitive deficits, potentially leading to better overall functional outcomes and quality of life for chronic stroke patients.

This study has certain limitations. Firstly, the absence of long-term follow-up prevents the evaluation of sustained cognitive and neurophysiological changes, so further studies should be conducted to assess the durability of improvements. Secondly, although the MoCA score range of 18 to 25 was used as an inclusion criterion, a comprehensive assessment of baseline cognitive characteristics was not conducted for both groups. Future studies should incorporate detailed cognitive profiling to better understand individual variability in response to the intervention. Thirdly, while QEEG was used as an objective measure of neuroplasticity, future research should consider incorporating multimodal neuroimaging techniques, such as fMRI or PET scans, to further validate cortical reorganization findings. Moreover, the relatively small sample size may restrict the generalizability of the findings, emphasizing the need for larger-scale studies to enhance the reliability and external validity of the results. Lastly, stroke severity was not assessed, so this assessment should be considered in further research.

## 5. Conclusions

In summary, our findings demonstrate that integrating cognitive–behavioral training with a selected physical therapy program significantly enhances cortical reorganization and cognitive performance in chronic stroke patients. The observed improvements in QEEG, MoCA, and RehaCom measures indicate that this combined approach may more effectively address cognitive deficits. Future research should focus on enhancing intervention protocols and evaluating long-term outcomes to further validate and optimize this integrated rehabilitation strategy.

## Figures and Tables

**Table 1:** Comparison of subject characteristics between study and control groups.

	Study Group	Control Group	*p*-Value
Age (years)	55.53 ± 4.71	54.13 ± 3.54	0.72
Sex, n (%)			
Females	1 (6.7%)	2 (13.3%)	0.54
Males	14 (93.3%)	13 (86.7%)	
Affected side			
Right side	2 (13.3%)	3 (20%)	0.62
Left side	13 (86.7%)	12 (80%)	

*p*-value: probability value, %: percentage, and n: number.

**Table 2:** Mean QEEG results pre- and post-treatment for the study and control groups.

	Pre-Treatment	Post-Treatment			
	Mean ± SD	Mean ± SD	MD	% Change	*p*-Value
F3					
Study group	5.21 ± 0.28	5.92 ± 0.24	−0.71	13.63	0.001
Control group	5.17 ± 0.44	5.49 ± 0.41	−0.32	6.19	0.009
MD	0.04	0.43			
*p*-value *	0.79	0.002			
F4					
Study group	5.69 ± 0.27	6.23 ± 0.36	−0.54	9.49	0.001
Control group	5.58 ± 0.29	5.81 ± 0.45	−0.23	4.12	0.01
MD	0.11	0.42			
*p*-value *	0.27	0.01			
T5					
Study group	5.47 ± 0.46	6.26 ± 0.42	−0.79	14.46	0.001
Control group	5.45 ± 0.58	5.91 ± 0.44	−0.46	8.43	0.003
MD	0.02	0.35			
*p*-value *	0.93	0.03			
T6					
Study group	5.72 ± 0.51	6.51 ± 0.46	−0.79	−13.81	0.001
Control group	5.87 ± 0.33	6.13 ± 0.36	−0.26	−4.43	0.007
MD	−0.15	0.38			
	0.32	0.01			

SD, standard deviation; MD, mean difference; *p*-value, within-group probability value. *p*-value *, between-group probability level.

**Table 3:** Mean MoCA pre- and post-treatment of the study and control groups.

	Pre-Treatment	Post-Treatment			
	Mean ± SD	Mean ± SD	MD	% Change	*p*-Value
MoCA					
Study group	19.20 ± 1.52	22.46 ± 1.35	−3.26	16.98	0.001
Control group	19.86 ± 1.30	21.33 ± 1.11	−1.47	7.40	0.001
MD	−0.66	1.13			
*p*-value *	0.21	0.01			

SD, standard deviation; MD, mean difference; *p*-value, within-group probability value. *p*-value *, between-group probability level.

**Table 4:** Mean logical reasoning, attention and concentration, memory level, reaction behavior level, and correct reactions pre- and post-treatment for the study and control groups.

	Pre-Treatment	Post-Treatment			
	Mean ± SD	Mean ± SD	MD	% Change	*p*-Value
Logical reasoning level					
Study group	3.13 ± 0.35	5.26 ± 0.79	−2.13	68.05	0.001
Control group	3.26 ± 0.45	3.73 ± 0.59	−0.47	14.42	0.02
MD	−0.13	1.53			
*p*-value *	0.37	0.001			
Attention and concentration level					
Study group	2.86 ± 0.64	5.66 ± 0.89	−2.80	97.90	0.001
Control group	2.93 ± 0.59	3.46 ± 0.83	−0.53	18.09	0.007
MD	−0.07	2.2			
*p*-value *	0.77	0.001			
Memory level					
Study group	2.73 ± 0.59	5.86 ± 0.92	−3.13	114.65	0.001
Control group	2.80 ± 0.77	3.53 ± 1.12	−0.73	26.07	0.01
MD	−0.07	2.33			
*p*-value *	0.79	0.001			
Reaction behavior level					
Study group	3.20 ± 0.41	5.20 ± 0.86	−2.00	62.50	0.001
Control group	3.33 ± 0.48	3.8 ± 0.67	−0.47	14.11	0.009
MD	−0.13	1.4			
*p*-value *	0.42	0.001			
Correct reactions					
Study group	3.46 ± 0.99	5.40 ± 0.91	−1.94	56.07	0.001
Control group	3.13 ± 0.83	3.67 ± 0.82	−0.54	17.25	0.01
MD	0.33	1.73			
*p*-value *	0.32	0.001			

SD, standard deviation; MD, mean difference; *p*-value, within-group probability value. *p*-value *, between-group probability level.

**Table 5:** Mean incorrect items, MRT, and missed items pre- and post-treatment for the study and control groups.

	Pre-Treatment	Post-Treatment			
	Mean ± SD	Mean ± SD	MD	% Change	*p*-Value
Incorrect items					
Study group	3.66 ± 0.72	1.13 ± 0.74	2.53	69.13	0.001
Control group	3.46 ± 0.74	3.00 ± 0.53	0.46	13.29	0.02
MD	0.2	−1.87			
*p*-value *	0.46	0.001			
MRT (msec)					
Study group	2095.2 ± 111.02	1250 ± 89.28	845.2	40.34	0.001
Control group	2109.66 ± 118.31	1824.6 ± 99.73	285.06	13.51	0.001
MD	−14.46	−574.6			
*p*-value *	0.73	0.001			
Missed items					
Study group	3.07 ± 0.70	1.07 ± 0.59	2	65.15	0.001
Control group	3.20 ± 0.86	2.13 ± 0.63	1.07	33.44	0.001
MD	−0.13	−1.06			
*p*-value *	0.64	0.001			

SD, standard deviation; MD, mean difference; *p*-value, within-group probability value. *p*-value *, between-group probability level.

**Table 6:** ANCOVA analysis of the RehaCom covariates.

Source	Dependent Variable	SS	df	MS	F Value	*p*-Value	Partial Eta Squared
Corrected Model	Attention and concentration pre level	42.707	9	4.745	9.529	0.000	0.811
	MRI level pre	2,412,998.657	9	268,110.962	58.908	0.000	0.964
	Memory level pre	55.093	9	6.121	6.521	0.000	0.746
	Missed item level pre	14.787	9	1.643	4.919	0.001	0.689
	Reaction behavior level pre	20.762	9	2.307	5.362	0.001	0.707
	Correct reactions pre	24.407	9	2.712	7.793	0.000	0.778
	Logical reasoning level pre	30.258	9	3.362	11.130	0.000	0.834
	Incorrect items pre	39.634	9	4.404	11.198	0.000	0.834
Intercept	Attention and concentration pre level	1.287	1	1.287	2.585	0.124	0.114
	MRI level pre	4648.275	1	4648.275	1.021	0.324	0.049
	Memory level pre	3.508	1	3.508	3.737	0.068	0.157
	Missed item level pre	0.034	1	0.034	0.101	0.754	0.005
	Reaction behavior level pre	0.792	1	0.792	1.841	0.190	0.084
	Correct reactions pre	2.611	1	2.611	7.502	0.013	0.273
	Logical reasoning level pre	0.201	1	0.201	0.666	0.424	0.032
	Incorrect items pre	0.012	1	0.012	0.031	0.863	0.002
Attention and concentration pre level	Attention and concentration pre level	0.013	1	0.013	0.026	0.874	0.001
	MRI level pre	3167.423	1	3167.423	0.696	0.414	0.034
	Memory level pre	2.113	1	2.113	2.251	0.149	0.101
	Missed item level pre	0.011	1	0.011	0.034	0.856	0.002
	Reaction behavior level pre	0.524	1	0.524	1.218	0.283	0.057
	Correct reactions pre	1.677	1	1.677	4.819	0.040	0.194
	Logical reasoning level pre	0.198	1	0.198	0.654	0.428	0.032
	Incorrect items pre	0.013	1	0.013	0.033	0.857	0.002
MRI level pre	Attention and concentration pre level	0.881	1	0.881	1.770	0.198	0.081
	MRI level pre	56,069.458	1	56,069.458	12.319	0.002	0.381
	Memory level pre	0.406	1	0.406	0.433	0.518	0.021
	Missed item level pre	0.136	1	0.136	0.406	0.531	0.020
	Reaction behavior level pre	0.289	1	0.289	0.673	0.422	0.033
	Correct reactions pre	0.432	1	0.432	1.242	0.278	0.058
	Logical reasoning level pre	0.064	1	0.064	0.211	0.651	0.010
	Incorrect items pre	0.024	1	0.024	0.060	0.809	0.003
Memory level pre	Attention and concentration pre level	1.678	1	1.678	3.369	0.081	0.144
	MRI level pre	3248.483	1	3248.483	0.714	0.408	0.034
	Memory level pre	2.154	1	2.154	2.294	0.145	0.103
	Missed item level pre	0.329	1	0.329	0.985	0.333	0.047
	Reaction behavior level pre	0.257	1	0.257	0.598	0.448	0.029
	Correct reactions pre	0.373	1	0.373	1.073	0.313	0.051
	Logical reasoning level pre	1.555	1	1.555	5.148	0.034	0.205
	Incorrect items pre	0.090	1	0.090	0.228	0.638	0.011
Missed item level pre	Attention and concentration pre level	0.021	1	0.021	0.043	0.838	0.002
	MRI level pre	33.803	1	33.803	0.007	0.932	0.000
	Memory level pre	0.275	1	0.275	0.293	0.594	0.014
	Missed item level pre	3.356	1	3.356	10.048	0.005	0.334
	Reaction behavior level pre	0.607	1	0.607	1.411	0.249	0.066
	Correct reactions pre	0.113	1	0.113	0.326	0.574	0.016
	Logical reasoning level pre	0.053	1	0.053	0.175	0.681	0.009
	Incorrect items pre	0.242	1	0.242	0.616	0.442	0.030
Reaction behavior level pre	Attention and concentration pre level	0.454	1	0.454	0.912	0.351	0.044
	MRI level pre	94.470	1	94.470	0.021	0.887	0.001
	Memory level pre	1.253	1	1.253	1.335	0.262	0.063
	Missed item level pre	0.698	1	0.698	2.089	0.164	0.095
	Reaction behavior level pre	0.007	1	0.007	0.017	0.899	0.001
	Correct reactions pre	0.938	1	0.938	2.696	0.116	0.119
	Logical reasoning level pre	5.222	1	5.222	17.287	0.000	0.464
	Incorrect items pre	0.011	1	0.011	0.029	0.866	0.001
Correct reactions pre	Correct reactions pre	0.775	1	0.775	1.557	0.226	0.072
	MRI level pre	467.993	1	467.993	0.103	0.752	0.005
	Memory level pre	0.128	1	0.128	0.136	0.716	0.007
	Missed item level pre	0.047	1	0.047	0.140	0.712	0.007
	Reaction behavior level pre	0.731	1	0.731	1.700	0.207	0.078
	Correct reactions pre	2.399	1	2.399	6.893	0.016	0.256
	Logical reasoning level pre	0.503	1	0.503	1.665	0.212	0.077
	Incorrect items pre	1.558	1	1.558	3.962	0.060	0.165
Logical reasoning level pre	Attention and concentration pre level	0.296	1	0.296	0.595	0.450	0.029
	MRI level pre	70.589	1	70.589	0.016	0.902	0.001
	Memory level pre	1.644	1	1.644	1.751	0.201	0.081
	Missed item level pre	0.299	1	0.299	0.895	0.356	0.043
	Reaction behavior level pre	0.046	1	0.046	0.106	0.748	0.005
	Correct reactions pre	0.186	1	0.186	0.534	0.473	0.026
	Logical reasoning level pre	4.107	1	4.107	13.597	0.001	0.405
	Incorrect items pre	0.267	1	0.267	0.680	0.419	0.033
Incorrect items pre	Attention and concentration pre level	0.103	1	0.103	0.208	0.654	0.010
	MRI level pre	3432.167	1	3432.167	0.754	0.395	0.036
	Memory level pre	0.167	1	0.167	0.178	0.678	0.009
	Missed item level pre	0.235	1	0.235	0.704	0.411	0.034
	Reaction behavior level pre	0.115	1	0.115	0.266	0.611	0.013
	Correct reactions pre	0.490	1	0.490	1.407	0.249	0.066
	Logical reasoning level pre	0.424	1	0.424	1.405	0.250	0.066
	Incorrect items pre	3.477	1	3.477	8.840	0.008	0.307
Group	Attention and concentration pre level	36.268	1	36.268	72.829	0.000	0.785
	MRI level pre	2,040,230.033	1	2,040,230.033	448.261	0.000	0.957
	Memory level pre	32.036	1	32.036	34.128	0.000	0.631
	Missed item level pre	6.963	1	6.963	20.846	0.000	0.510
	Reaction behavior level pre	15.388	1	15.388	35.768	0.000	0.641
	Correct reactions pre	12.088	1	12.088	34.736	0.000	0.635
	Logical reasoning level pre	20.813	1	20.813	68.899	0.000	0.775
	Incorrect items pre	27.000	1	27.000	68.654	0.000	0.774
Error	Attention and concentration pre level	9.960	20	0.498			
	MRI level pre	91,026.810	20	4551.340			
	Memory level pre	18.774	20	0.939			
	Missed item level pre	6.680	20	0.334			
	Reaction behavior level pre	8.604	20	0.430			
	Correct reactions pre	6.960	20	0.348			
	Logical reasoning level pre	6.042	20	0.302			
	Incorrect items pre	7.866	20	0.393			
Total	Attention and concentration pre level	136.000	30				
	MRI level pre	2,503,975.000	30				
	Memory level pre	186.000	30				
	Missed item level pre	92.000	30				
	Reaction behavior level pre	75.000	30				
	Correct reactions pre	77.000	30				
	Logical reasoning level pre	87.000	30				
	Incorrect items pre	115.000	30				

## Data Availability

The data from this study are available upon reasonable request from the principal investigator, due to privacy and ethical considerations.

## Abbreviations

The following abbreviations are used in this manuscript:

ADL	Activities of daily living
CBT	Cognitive–behavioral training
CT	Computed tomography
EEG	Electroencephalography
MoCA	Montreal Cognitive Assessment
MRI	Magnetic resonance imaging
PSCI	Post-stroke cognitive impairment
QEEG	Quantitative electroencephalogram
